# Precise intraoperative sentinel lymph node biopsies guided by lymphatic drainage in breast cancer

**DOI:** 10.18632/oncotarget.18624

**Published:** 2017-06-27

**Authors:** Xiaoyan Li, Sisi Chen, Liyu Jiang, Xiaoli Kong, Tingting Ma, Hong Xu, Qifeng Yang

**Affiliations:** ^1^ Department of Breast Surgery, Qilu Hospital, Shandong University, Jinan, Shandong 250012, P.R. China; ^2^ Department of Breast Surgery, General Hospital of Chinese Armed Police Force, Beijing 100039, P.R. China; ^3^ Department of Pathology Tissue Bank, Qilu Hospital, Shandong University, Jinan, Shandong 250012, P.R. China

**Keywords:** precise sentinel lymph node biopsy, trSLNs, breast cancer

## Abstract

The purpose of this study was to present a novel surgical method for intraoperative precise sentinel lymph node biopsy (SLNB) and to determine its clinical efficacy and sensitivity in breast cancer patients. The sentinel lymph nodes (SLNs) were preoperatively evaluated by axillary ultrasound. The intraoperative detection of SLNs was guided by lymphatic drainage pathway. The lymphatic vessels and SLNs were visualized. During operation, we searched for all the true SLNs (trSLNs), para-SLNs (paSLNs) and post-SLNs (poSLNs) followed lymphatic drainage ducts. After precisely locating the lymphatic channels and lymph node, all the lymph nodes that firstly receive lymphatic drainage are designated as trSLNs. We precisely distinguished the trSLNs, paSLNs and poSLNs. We found the average number of trSLNs ranged from1 to 6. In addition, we assessed the novel technique in a total of 125 breast cancer patients. trSLNs were successfully identified in all patients (detection rate: 100 %). The accuracy of trSLNs is 99.2%. Data from our study strongly suggest that our method is a feasible and effective for the detection of precise trSLNs in breast cancer with real-time observations. (ClinicalTrials.gov number, NCT02651142).

## INTRODUCTION

Sentinel lymph nodes (SLN) are the first lymph nodes that receive lymphatic drainage. Cancer cells are mainly spread through the lymphatic system in early stage of breast cancer. Regional lymph node status is one of the strongest prognostic factors for primary breast cancer. Therefore it is important to exactly identify positive lymph nodes for axillary management because false-negative results will lead to inaccurate assessment of regional lymph node and inadequate therapeutic strategies. Axillary lymph node dissection has been used to evaluate lymph node status. However, it appears correlated with higher morbidity of lymphedema, arm pain and stiffness, seroma formation. At present sentinel lymph nodes biopsy (SLNB) has been used successfully for axillary evaluation in breast cancer and it's considered the standard method for early-stage breast cancer without clinically or radiologically axillary lymph node metastases [1, 2]. The American Society of Clinical Oncology recommend the minimum sentinel node identification rate is 85% and the false-negative rate is less than 5% before abandoning the axillary dissection [3]. Studies shown that the dual technique involving injection of radioisotope and blue dye into either the interstitial breast tissue around the tumor or the periareolar tissue are common methods for SLN biopsy [4]. Also it is possible to use each one independently. Though many new techniques, such as Indocyanine green (ICG), have been employed for identifying SLN [5, 6], the specificity and risk of false-negative rate in mapping SLNs are still the main concern.

Today there are still many debates involving SLN biopsy. One is the exact number of SLNs for patients. Studies showed that patients may have different types of sentinel lymph channels and different numbers of SLNs [7, 8]. As the drainage of lymph nodes are in multiple directions from the primary tumor, the type of sentinel lymph channels could affect the detection rate of SLN biopsy. This could partly explain that the false-negative rate of SLN failed to be achieved even for experienced surgeons. Previous studies have found that the number of SLNs removed has a statistical effect on the false-negative rate [9, 10]. In many studies, lymph nodes that contained ICG fluorescence, radioactivity and/or blue staining were usually defined as SLNs and excised by surgeons [11–13]. Studies have reported that the SLNs ranged from 1 to 12 for patients in many countries including China, Germany, Poland, USA and Netherlands [14], and this could not be explained by races. As the number of SLNs for one patient is fixed, we probably removed more lymph nodes that are not true SLNs in order to reduce the false-negative rate clinically. The overtreatment is even more common in post-neoadjuvant chemotherapy. Boughey et al. [15] reported that removing at least two sentinel nodes could lower the false-negative rate of SLNs in post-neoadjuvant chemotherapy. The similar result was reported by Boileau and his colleges [16]. However, if only one true SLN actually exist in patients, excess axillary lymph node that are not true SLNs will be dissected. On the other hand, when the true SLNs are not fully dissected, the remaining SLNs maybe positive and the patients may have increased local recurrence and poor prognosis due to the false-negativity of SLNB. Therefore, other methods for increasing the sentinel lymph node identification rate and reducing the false-negative rate are welcome to be explored. In this study, we present a novel surgical method that enables the precise identification of trSLNs guided by lymphatic drainage pathway in breast cancer.

## RESULTS

### Precise sentinel lymph node biopsy

**Figure 1:**
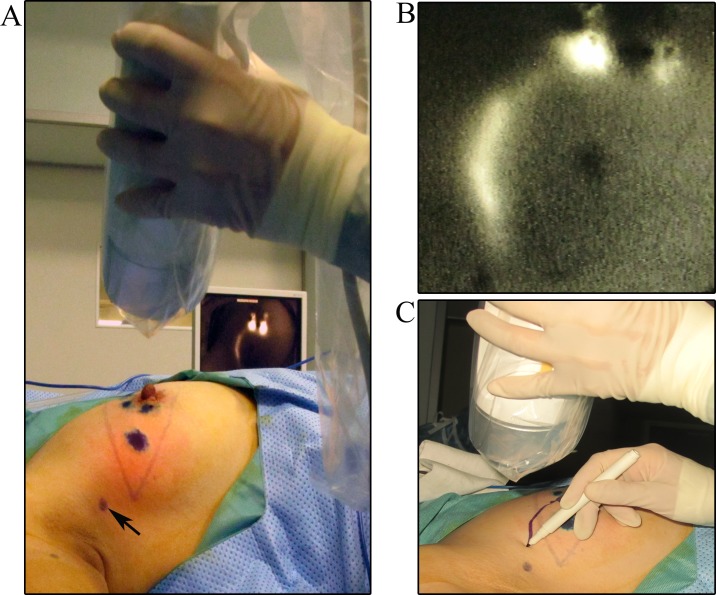
Marking the whole lymphatic vessels guided by ICG fluorescence **(A)** The indocyanine green fluorescence real-time dynamic imaging system. Black arrows were the possible SLNs under preoperatively axillary ultrasound assessment. **(B)** ICG fluorescence image of lymphatic vessels visualized in real time on the monitor screen of the dynamic imaging system. **(C)** Drawing the lymphatic drainage pathways guided by ICG fluorescence.

**Figure 2:**
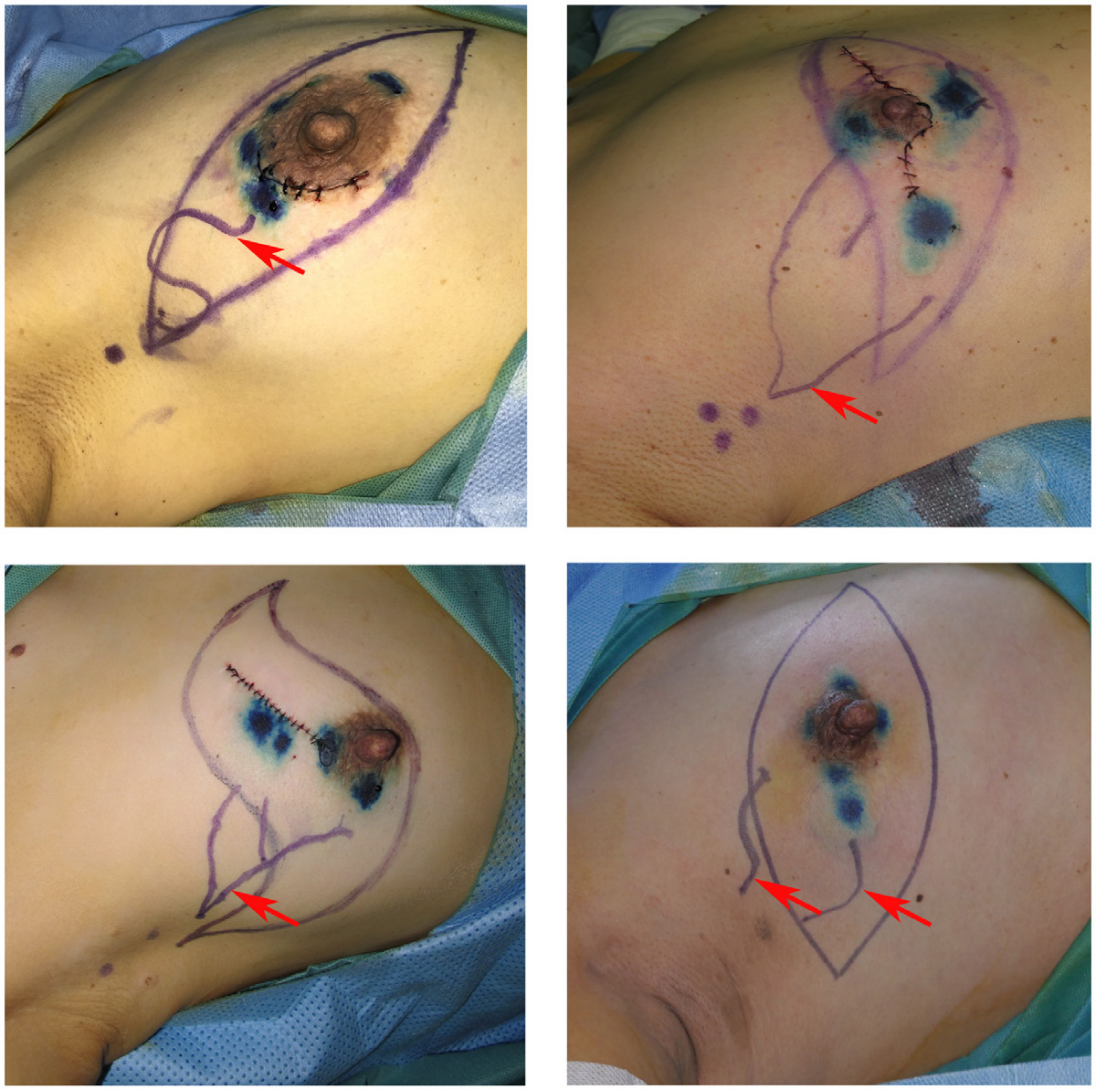
Represented images of marked lymphatic drainage pathways guided by ICG fluorescence Red arrows were the lymphatic channels.

**Figure 3:**
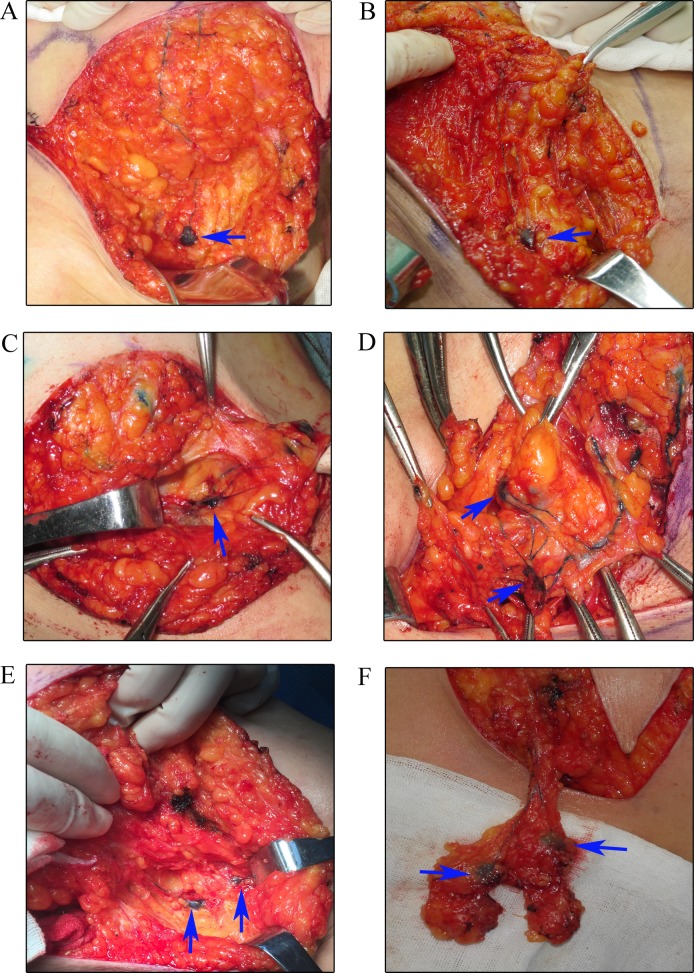
Identification the trSLNs with various input lymphatic ducts **(A)** trSLN with one input lymphatic duct. **(B)** trSLN with one input lymphatic duct. **(C)** trSLN with three input lymphatic ducts. **(D)** trSLN with multiple input lymphatic ducts. **(E)** SLNs with two parallel input lymphatic ducts. **(F)** trSLNs with bifurcated input lymphatic vessels. Blue arrows were SLNs.

**Figure 4:**
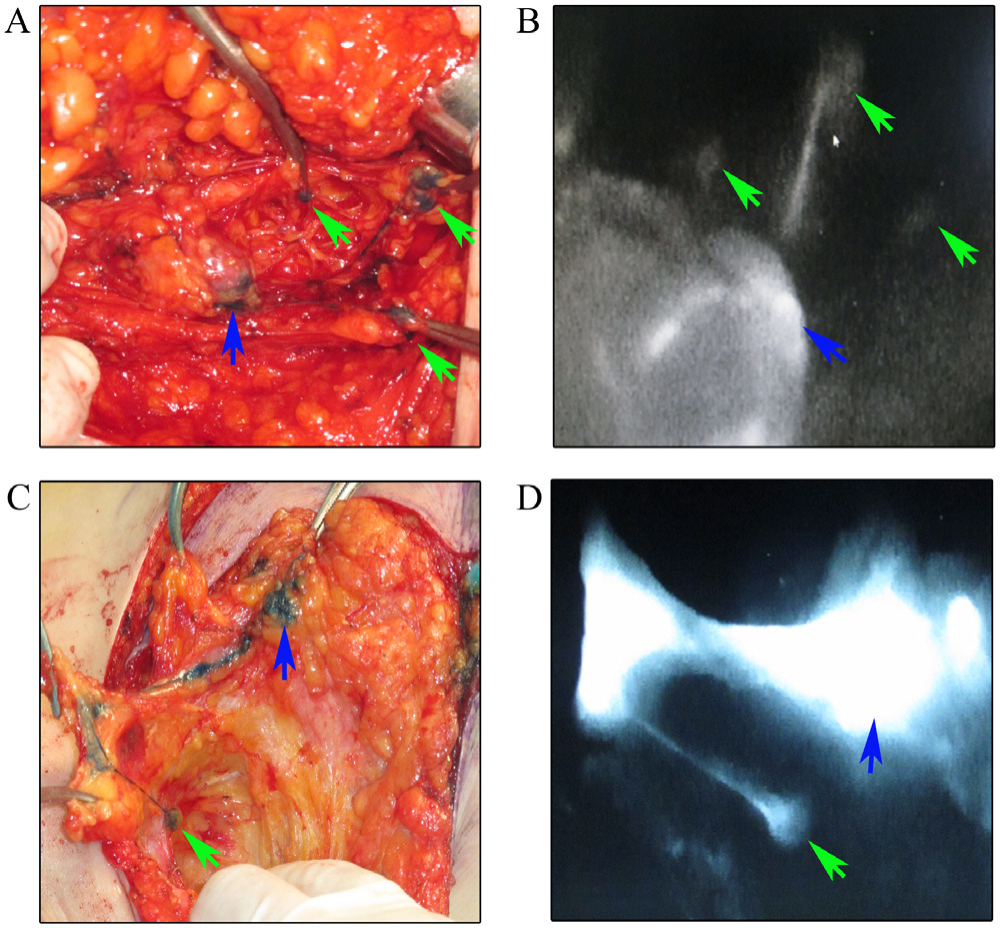
Identification the trSLNs with poSLNs **(A)** Dissection the whole lymphatic vessels and trSLN with three poSLNs. **(B)** The shiny fluorescent images of A on the monitor screen of the dynamic imaging system. **(C)** Dissection the whole lymphatic vessels and trSLN with one poSLN. **(D)** The shiny fluorescent images of C on the monitor screen of the dynamic imaging system. Blue arrows were trSLNs and green arrows were poSLNs.

**Figure 5:**
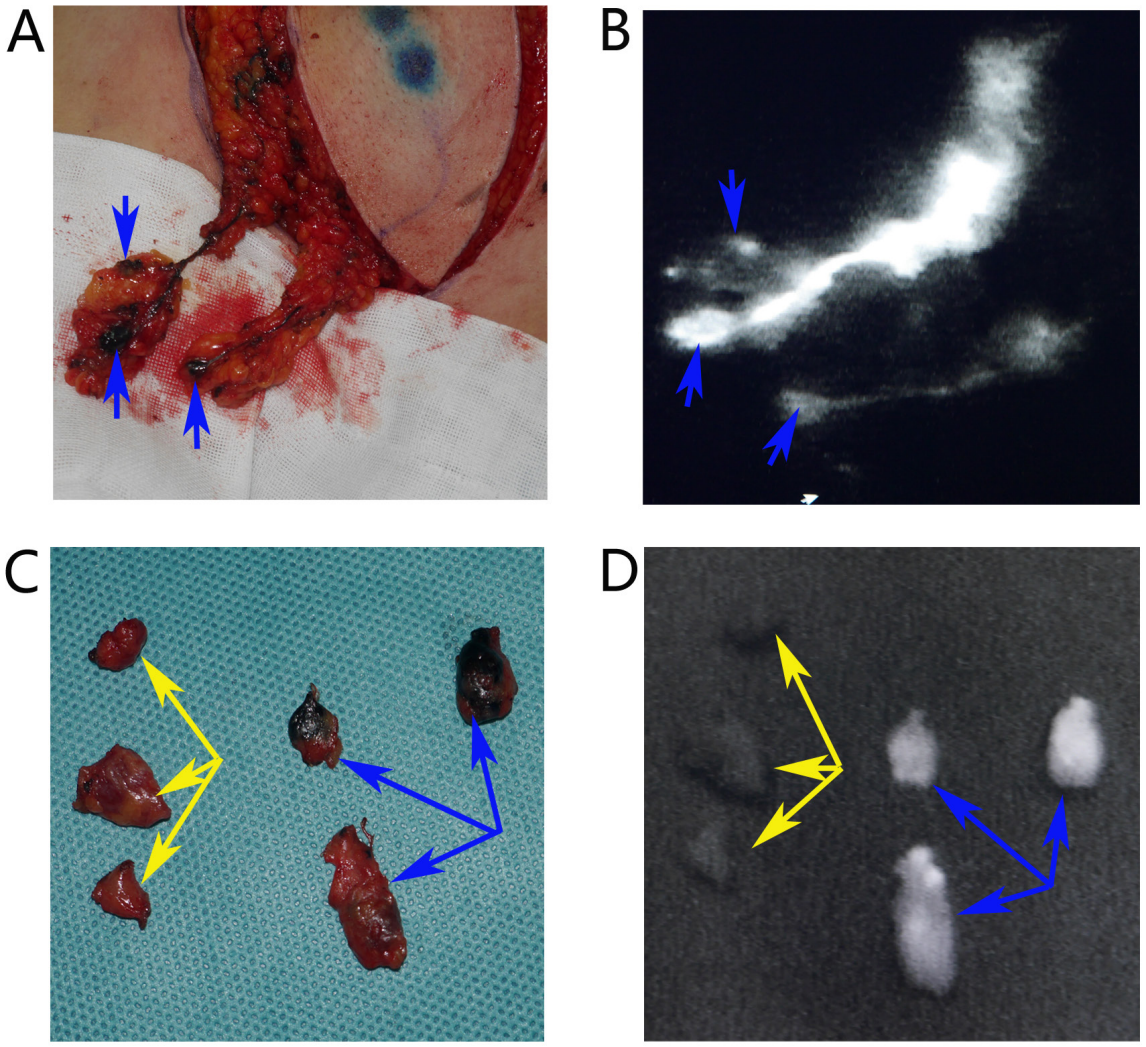
Identification the trSLNs with paSLNs **(A)** Dissection the whole lymphatic vessels and trSLNs. **(B)** The shiny fluorescent images of A on the monitor screen of the dynamic imaging system. **(C)** All the resected trSLNs and paSLNs. **(D)** The shiny fluorescence images of all trSLNs and paSLNs. Blue arrows were trSLNs and yellow arrows were paSLNs.

All patients received axillary ultrasound and all the possible sentinel lymph nodes were preoperative assessed and marked on the skin (Figure 1A). In our study, we injected methylene blue into the periareolar tissue in multiple deposits directly before surgery. Five minutes later, ICG were injected in the same position. Then the surgical field was illuminated using a near-infrared illumination system for ICG lymphography (Ming De Fluorescence Imaging System). As ICG fluorescence illumination can be captured to a depth of less than 2 mm, lymph flow over the breast skin surface and in the superficial tissues can be visualized. Guided by the ICG fluorescence real-time dynamic imaging system, the shiny lymphatic vessels could be observed transcutaneously in real time on the monitor screen (Figure 1A and 1B). We next marked the lymphatic drainage pathways with a pen so that we don't disrupt the lymphatic channels during surgery (Figure 1C). Represented images of marked lymphatic drainage pathways guided by ICG fluorescence were shown in Figure 2. As the whole lymphatic vessels were marked on skin, we incised the skin and fully dissect out the lymphatic vessels guided by both fluorescence and blue stained lymphatic duct. The lymph nodes that accumulated methylene blue stained blue could be observed with the naked eye while that accumulated ICG appeared as shiny fluorescent spots. After incision of skin, the lymphatic drainage pathways and SLNs were observed apparently. The lymph nodes were stained by both ICG and methylene blue while others exhibit only green staining or blue staining. Then we searched for SLNs directly guided by lymphatic drainage pathway and exactly dissected out the lymphatic vessels and lymph nodes. In clinical practice, we found that the time interval after injection methylene blue and ICG can affect the staining of lymph nodes. If the time is not enough, the SLNs may not fully identified especially for SLNs with more than one lymphatic ducts. Otherwise, the non-SLNs were also stained. Our time interval was about15 minutes after injection ICG. After precisely locating the lymphatic channels and lymph node, all the green staining and blue staining lymph nodes that firstly receive lymphatic drainage are designated as trSLNs. Our finding showed that different trSLNs had different number of input lymphatic ducts. Some had one input lymphatic duct while others had two or more ducts (Figure 3). What's more, we found that some lymph nodes that directly followed the trSLNs could be blue stained and had fluorescence. We defined that lymph node as post-SLNs (poSLNs) (Figure 4). If we did not dissected out and marked all the lymphatic drainage pathways. We may remove the poSLNs as trSLNs. In addition, we found many swollen lymph nodes closed to trSLNs and we defined these as para-SLNs (paSLNs). They weren't stained neither by fluorescence nor methylene blue dye (Figure 5). These maybe also be removed by surgeons clinically. If so the SLNB for these patients should be more carefully and remove the trSLNs without poSLNs or paSLNs in clinical practice.

### Evaluation on clinical application of SLNB

In order to determine the clinical efficacy and sensitivity of our method in breast cancer patients, 125 breast cancer patients were enrolled in our clinical trials for verification. The patients were diagnosed as invasive ductal carcinoma or ductal carcinoma *in situ* or invasive lobular carcinoma. Axillary lymph node dissection was performed in all the patients so we could precisely evaluation the status of locoreginal lymph node. The average age of patients was 49.47 years (range, 31–78 years). None of the patients experienced severe adverse reactions or complications that were related to the intraoperative injection of ICG combined with methylene blue. It's exciting that we identified all the trSLNs by our novel surgical navigation method (detection rate: 100 %). A total of 42 patients have 1 trSLN, 41 patients have 2 trSLNs, 19 patients have 3 trSLNs, 14 patients have 4 trSLNs, 4 patients have 5 trSLNs, 5 patients have 6 trSLNs. The average number of trSLNs was 2.3 (range 1–6 trSLNs) in all patients. In our practice, about 45.6% (57/125) patients had positive SLNs while the other 68 were negative. A total of 57 patients were trSLNs positive. 1 case was ductal carcinoma *in situ* with microinvasion, 1 case was invasive lobular carcinoma and 55 cases were invasive ductal carcinoma. Among these trSLNs positive 57 patients, 31patients have no additional metastatic axillary lymph node. However, in the 58 trSLNs negative patients, only one had metastatic lymph node in the dissected axillary lymph node. The false negative rate is 1.7% in our study. The accuracy of our data is 99.2%. By further analyzing the data, we found this patient had four tumors in breast. Therefore, the lymphatic vessels may be disrupted when excision the tumors for biopsy. The positive lymph node probably was a true SLN that we did not identify. This should be further confirmed in the following study.

## DISCUSSION

SLNB has been adopted as a standard method for axillary nodal staging by most surgeons [17]. At present, a blue dye or a radioactive tracer, and/or a combination of these two methods are the most performed techniques for SLN mapping and identification [13, 18]. Large randomized trials showed that the overall accuracy SLNs was 93% to 97% and a false negative rate was about 9.8% [19–21]. The SLN identification rate is lower and false negative rate is higher in patients receiving post-neoadjuvant chemotherapy. Clinical trials reported a higher false negative rate when only one SLN was found [22]. Kennedy reported that removal of two sentinel nodes significantly reduced the false-negative rate compared with removal of one node [23]. Other studies suggested that four might be an optimal threshold number of SLNs to be removed [24, 25]. Aoyama and Chi showed that the detection rate was 100% when they removed 1∼12 SLNs [26, 27].

How many SLNs should be resected? As SLNs are the definition of anatomy and patients have different types of sentinel lymph channels and different numbers of SLNs, so in our opinion we couldn't define the number of SLNs that should be removed. On the contrary, we should dissect all the true SLNs. Though alternative techniques for increasing the SLNs accuracy rate and reducing the false-negative rate need to be developed, the most important is the method used to identify the true SLNs in clinical practice. As we all know axillary ultrasound can identify the status of axillary lymph nodes based on sizes and morphology of the lymph nodes and it's considered as a noninvasive technique for staging of the axilla [28, 29]. In the present study, we firstly evaluate the status by axillary ultrasound, and we localized the suspected metastatic lymph node. Then we did precise SLNB followed by lymphatic drainage pathway with the help of ICG fluorescence and methylene blue dye. The surgical procedure could be used to precisely locate SLNs and the lymphatic vessels. Then we exactly dissect out all the lymphatic vessels and SLNs for patients receiving mastectomy. For patients receiving breast conservation surgery, the ICG fluorescence image of lymphatic vessels can be visualized on the skin surface, and the location of the skin incision for the detection of SLNs can be designed intraoperatively at the end of the lymphatic vessels. Then the lymphatic drainage ducts could be dissected out. And we removed the trSLNs guided by fluorescence and blue stained lymph nodes. What's more important, we could distinguish between the trSLNs, paSLNs and poSLNs. In many studies, surgeons may remove the paSLNs and poSLNs and defined as trSLNs. By using our method, we could easily and exactly identify the trSLNs rather than paSLNs and poSLNs. We found that the average number of trSLNs ranged from 1 to 6 in breast cancer patients. In addition, our clinical practice further demonstrated the feasibility and accuracy of our method for precise SLNB in breast cancer. Our data suggest that the novel technique could not only provide the accurate location of trSLNs and lymphatic mapping, but it also be used to successfully remove the trSLNs with a high sensitivity and accuracy. Therefore, this method potentially provides an alternative for the exact SLNB in breast cancer. However, our present study still had some limitations. First, the number of patients is relatively small. Second, there is no prognosis and complications information for patients. In addition, early breast cancer is treated usually by breast conserving surgery the SLNB. We should explore easier methods for identifying trSLN in these cases with small incisions. Therefore a large randomized, controlled trial is needed in the future.

In conclusion, our data have demonstrated that our method exhibited a very high sensitivity as well as lower false negative rate. Also it could provide a more accurate detection of the true SLNs clinically. With the novel method, we could easily identify the true SLNs precisely and it will provide important information to guide precise treatment decisions for breast cancer patients in the future.

## MATERIALS AND METHODS

### Patients

A total of 125 breast cancer patients were enrolled. All the patients were received SLNB firstly, and then followed by axillary lymph node dissection (ALND) between September 2015 and Auguest 2016 in Qiu Hospital of Shandong University. Inclusion criteria were a histopathological diagnosis of breast cancer, and an indication for mastectomy or breast conservation therapy without a clinically positive lymph node status. Exclusion Criteria were fine needle aspiration cytology was positive patient. The detailed clinical data and tumor characteristics are summarized in Table 1. After precisely locating the lymphatic channels and lymph node, all the lymph nodes that firstly receive lymphatic drainage are designated as trSLNs. The lymph nodes that directly followed the trSLNs were defined as post-SLNs (poSLNs). These swollen axillary lymph nodes around the trSLNs were defined as para-sentinel lymph nodes (para-SLNs). Written informed consent was obtained and our project was approved by Ethics Committee on Scientific Research of Shandong University Qilu Hospital. A histopathological assessment of the resection specimens were conducted postoperatively.

**Table 1:** Clinicopathological features of the patients and tumors

Characteristics	Number
**Age**	
≤ 45	48
>45	77
**Histologic subtype**	
Invasive ductal carcinoma	109
Invasive Lobular carcinoma	2
Others	14
**Number of tumors**	
1	118
2	3
3	3
4	1
**Tumor size**	
≤ 2cm	68
> 2cm	57
**ER status**	
Negative	36
Positive	89
**PR status**	
Negative	40
Positive	85
**Her-2 status**	
Negative	76
Positive	49

ER: Estrogen Receptor; PR: Progesterone Receptor;

Her-2: human epidermalgrowth factor receptor-2.

### Statistical analyses

The accuracy and false-negative rate were calculated by comparing the results of the SLNB and the histopathology of the resection specimens in axillary lymph node dissection. False negative rate = number of false negative SLNs/(true positive + false negative nodes) x 100. Accuracy = (true positive + true negative nodes)/total nodes x 100.
